# Advancing Infodemiology in a Digital Intensive Era

**DOI:** 10.2196/37115

**Published:** 2022-02-14

**Authors:** Tim Mackey, Cynthia Baur, Gunther Eysenbach

**Affiliations:** 1 Global Health Program Department of Anthropology University of California, San Diego La Jolla, CA United States; 2 Global Health Policy and Data Institute San Diego, CA United States; 3 Horowitz Center for Health Literacy University of Maryland School of Public Health College Park, MD United States; 4 JMIR Publications Toronto, ON Canada; 5 Health Information Science University of Victoria Victoria, BC Canada

**Keywords:** infoveillance, infodemiology, infodemic, social listening, COVID-19, Twitter, machine learning, surveillance, infoveillance, public health, infodemic management, fake news, conspiracy, health communication, medical informatics, digital health, scholarly publishing, misinformation, digital epidemiology, medical informatics, journalogy

## Infodemiology: Then and Now

### Origins of Infodemiology

The concept of *infodemiology* was introduced in 2002 by Gunther Eysenbach [1], the editor and founder of the *Journal of Medical Internet Research* (*JMIR*), to identify, characterize, and measure misinformation, in analogy to epidemiology, the science of determinants and distribution of disease:

A new research discipline and methodology has emerged—the study of the determinants and distribution of health information and misinformation—which may be useful in guiding health professionals and patients to quality health information on the Internet.

1

Having done research on how to quantify and prevent outbreaks of misinformation [2,3], Eysenbach [4] was acutely aware that “quality of health information” and “misinformation” were elusive concepts with little or no consensus on how to define, let alone combat low quality and misinformation. For these reasons, the original definition of “infodemiology” purposefully avoided the term “misinformation.”

Information epidemiology, or infodemiology, identifies areas where there is a knowledge translation gap between best evidence (what some experts know) and practice (what most people do or believe), as well as markers for “high-quality” information.

1

In subsequent studies, Eysenbach [5,6] and the early work of others [7,8] found further use cases for studying information patterns and information retrieval patterns, including the detection of emerging outbreaks by studying the search and click behavior of populations [5]—ideas that were later adopted and implemented on a larger scale by Google Flu Trends [9].

The concept and area of study continued to evolve and advance, and by 2009, the now most frequently cited definition for infodemiology emerged in an article also by Eysenbach [10] in *JMIR*:

the science of distribution and determinants of information in an electronic medium, specifically the Internet, or in a population, with the ultimate aim to inform public health and public policy.

10

More than a decade since infodemiology entered the scientific consciousness, JMIR Publications has been committed to spearheading, advancing, and shaping this emerging field. The JMIR family of journals have strived to publish leading-edge studies that complement and push the methodological and disciplinary boundaries of health informatics research. Reflecting those efforts, a recent scoping review by Mavragani [11] found that more than 83% of studies focused on infodemiology and infoveillance have been published by JMIR Publications, with interest and number of publications increasing every year. Hence, recognizing a need for a formal scientific space to further catalyze advancement of this interdisciplinary community, in mid-2021, we launched JMIR Infodemiology.

### The Urgency of Now

Nearly 20 years after the concept was first introduced and with an increasing breadth and depth of research [11], infodemiology was further recognized as a critical field of study and formal practice in conjunction with the current COVID-19 pandemic. In March 2020, the World Health Organization (WHO) declared an “infodemic” and now defines it as when there is “too much information including false or misleading information in digital and physical environments during a disease outbreak” [4,12], and in June 2020, the WHO held its first WHO Infodemiology Conference [13], following a preparatory online crowdsourcing process to develop a policy framework to fight infodemics, which was published in *JMIR* [4,14].

The WHO would use this occasion to define infodemiology as the “science of managing infodemics” in the context of the COVID-19 infodemic itself, which aligns with the WHO’s important work and capacity building in advancing the field of infodemic management. These efforts include supporting the generation of tools to respond to misinformation, building community resilience to misinformation, fostering partnerships among multiple stakeholders (including the United Nations [UN] system, the technology sector, media, and civil society), and advocating for the issue through UN and WHO resolutions and community outreach and training [12,14]. These efforts have helped the concept of infodemiology gain traction among policy makers and public health professionals alike, though the scope of infodemiology is broader than a singular focus on managing infodemics.

Importantly, the convergence of factors—volume and speed of information, misinformation, and disinformation flow combined with political polarization [15-18]—makes the goal of forging a community of evidence-based practice for infodemic management one that we share as well. Supporting these shared goals of advancing the science of infodemiology; ensuring broad dissemination and translation of research; and, most importantly, pursuing science-based advocacy, findings from the WHO’s first Infodemiology Conference were published in the inaugural issue of JMIR Infodemiology [19]. In a relatively short period of time, JMIR Infodemiology has published several papers that address urgent needs of the COVID-19 infodemic (including studies addressing information demand and behavior [20], leveraging social listening across multiple data sources and languages [21], using mixed methods approaches blending online and offline data [22], and large-scale big data studies examining misinformation narratives [23] to name a few).

Crucially, though vaccines, public health interventions, and other medical countermeasures may ultimately lead to the halt or mitigation of the COVID-19 spread, the infodemic generated by this global pandemic will persist and mutate into new topics and opportunities for disinformation/misinformation in other health spaces. Who generates and shares information, how they use or share it and to what end, and how people respond to and contest information is already shaping the contours within which everyday health decisions, as well as the next public health emergency, are likely to occur. Although the COVID-19 pandemic is not the first instance in which digital media fueled global, national, and local struggles to define an information ecosystem [6], COVID-19’s residue along with the broad-ranging promise this field holds is an appropriate basis for a new multidisciplinary effort to foster and report on the science of infodemiology.

## Shaping the Field: Goals and Key Themes of Infodemiology

### Next Gen Supply and Demand

Eysenbach introduced the concepts of supply- [1] and demand-based [5] infodemiology that continue to provide a framework for identifying and exploring novel methods and applications of infodemiology and infoveillance [10]. The construct of supply-based infodemiology may be increasingly more robust than the earliest days of conceptualization, when supply focused more on *what* was published, especially measuring for or analyzing the quality of health information. Today, supply-based infodemiology could have as much focus on *how* information is published, republished, translated, and adapted, all with the need for a reflexive understanding of the sociocultural dynamics of influence and trust as well as the technical factors of communication timing, real-time monitoring, and automated responses or adaptations, among other variables. Public health communication may never have been a one-and-done effort, but with the pace of information transmission and potential distortion of that information (eg, in the case of the current COVID-19 infodemic), the evolving practice of information-sharing will continue to be an especially dynamic research area.

Demand-based infodemiology methods and applications have also become increasingly varied and more robust. Search and click measures continue to offer baseline insights, and yet more sophisticated capturing of a user’s entire journey on the internet or through smartphone searches and apps offers myriad ways to explore and track information-seeking behaviors. Regardless of the evolution of supply and demand methods, the ongoing need for novel methods for consumer and public health informatics to measure the epidemiology of information, describing and analyzing health information and communication patterns in “electronic media,” remains. What is more, we invite social and behavioral scientists to interpret measurements in different ways, further exploring the sociocultural and political dimensions that we may have otherwise tried to control for in the past but now acknowledge that we must engage with to make better sense of the variety of knowing-doing behavior patterns and gaps.

### Goals of Infodemiology

Earlier studies in the field of infodemiology include articles that set up early infoveillance systems and argued that public health agencies should prepare for the next pandemic by implementing social listening and media monitoring tools into pandemic preparedness plans, often framed in the context of syndromic surveillance [6,7,9]. Many of these applied infodemic concepts were tested to track misinformation and communication patterns on social media during the 2009 H1N1 pandemic, which was the first pandemic in the age of social media [6]. The idea that social media communication patterns may be predictive for other events was also used in the establishment of altmetrics to measure the uptake and knowledge translation of scientific work [24].

Since these early works that set the foundation of infodemiology research, the field has grown in data sources, methodologies, and practitioners. Despite these gains, deficits remain in maturing infodemiology into a more diverse, inclusive, and truly interdisciplinary field of practice, most of which focuses on the need for greater diversity of data sources, better triangulation of infodemiology insights from novel sources of online and offline information, and the need for greater inclusion of other health challenges that have traditionally been neglected or overlooked. One critical challenge to ensuring advancement of the field is bridging pre-existing disciplinary silos, where much of the innovation in methods, experimentation, and evaluation of data science approaches (eg, data mining, natural language processing, and forms of machine learning) still occur in the computer science (ACM) and engineering (IEEE) literature. However, this literature is often limited in its translation of research to real-world public health application, with studies in the social science (JSTOR, SSRN) and health and life sciences (PubMed-indexed articles) often filling this gap but inherently less technically rigorous. For example, studies of algorithmic bias, eHealth literacy, and cultural influences on online health information seeking are some areas where data and social science researchers can more actively collaborate to address existing gaps.

More generally, Mavragani’s [11] review discovered that the most popular data sources for infodemiology research are social media, search queries, websites and internet platforms, and mobile apps. The most frequently studied topics included epidemics, infectious diseases, flu, HIV/AIDS, measles, and other outbreaks, with drugs, tobacco and marijuana use, depression, suicide, cancer, and chronic disease also receiving attention [11]. Hence, the tracking of misinformation is but one line of infodemiology research, and harnessing the study of information patterns in cyberspace for other critical research questions is another, also envisioned in 2009 [7]. Additional findings from this review highlighted the following issues that need to be addressed for their implications to the field:

- Twitter and Google dominate as research data sources
- The need to take into account demographic differences in social media channel preferences
- The concentration of specific topics that may or may not represent people’s everyday health concerns and the most common causes of illness, injury, and death

This also requires us to ask more critical questions that advance and shape health policy and practice, and further extend the application of infodemiological principles to domains beyond health. Infodemiology has several advantages to that end. Web-based data makes data access and analysis faster than traditional research methods and at scale. It is also possible to retain anonymity while researching broad and distinct populations, though many ethical principles of conducting research using online data are still maturing [25]. It is also critical to make more intentional connections between information-seeking actions and real-world behavior through innovative study designs, such as using digital mixed methods approaches. Responding to these needs, JMIR Infodemiology aims to also explore infodemiology’s current limitations, such as research driven by current events and perpetuating population and channel biases, among others.

We see a unique opportunity, therefore, to accelerate the coordination of this broad-ranging research to greater effect on public health policy and practice and other pressing global issues. Our specific goals for the journal are to:

- Share what researchers from different disciplines are doing in how they collect and analyze online data
- Understand what online data say about offline human behavior
- Provide an intellectual space for researchers from different disciplines to cross theoretical and methodological boundaries
- Provide equal attention to supply-side and demand-side dynamics in an information-abundant global society
- Innovate and advance research design and practice by trialing and sharing new data sources and methods including through development of unique scientific content types and interaction tools
- Highlight biases, inequities, and limitations of digital spaces and claims about online behaviors

Key themes and research aims we will support, some of which we expound on briefly in the following section, will include exploring the supply and demand infodemiology framework; expanding and diversifying data sources; broadening the range and scope of global health challenges studied; supporting a multidisciplinary approach to infodemiology; supporting exploration of a range of analytical methods that can be applied to infodemiology; studying eHealth literacy and its connection to infoveillance; exploring population, algorithmic, descriptive, or sociocultural biases; and addressing issues relating to health equity.

### Infodemiology in a Digital Intensive Era

With over half of the world’s population currently on the internet and other connected devices, information access and availability can seem ubiquitous, while public opinion polls show many people are confused, unsure, or disbelieving of official information sources [26]. An increase in information and communication technology (ICT) access has led to a proliferation of data sources, including the evolution of the internet from web 1.0 (static websites and the dot-com boom) to 2.0 (software applications built on the web and the rise of social media and other interactive digital platforms), moving toward 3.0 (emergence of the semantic web, artificial intelligence imbedded on the web, and decentralized and distributed applications), and now discussions of a metaverse (generally the connection of networks of cyberspace and virtual worlds focused on social connection). The opportunity to leverage these data sources to improve individual and population health is now paramount, but equally important is ensuring that these sources do not lead to offline health harms or harms within digital communities themselves.

The expansion and evolution of the ICT ecosystem provides new opportunities to characterize and assess changes in large-scale human behavior. Understanding how data and behavior interact also requires recognition of the complex interplay between how people now increasingly rely on and are influenced by information exposure on these digital platforms that are increasingly primary sources of communication and health seeking behavior. Key pillars of infodemiology remain its ability to analyze (nowcast) and predict (forecast) forms of health behavior, diseases (especially those with behavioral risk factors), and epidemics, and to generate insights closer to real time; though as mentioned, the scope of the field is rapidly expanding, and other data sources such as digital biomarkers, including data from wearables, may enhance our ability to measure information and communication patterns for public health purposes [11].

As exponential growth in data generation and access has catalyzed the infodemiology field, the breakneck pace of successive complex global public health emergencies (including the 2003 severe acute respiratory syndrome outbreak, the 2009 H1N1 pandemic, the 2015 Zika outbreak, multiple Ebola virus outbreaks, the ongoing antimicrobial resistance crisis, the current opioid public health emergency, and the current 2019 COVID-19 global pandemic as a few examples) further necessitates its maturation to meet 21st century health challenges that the world now faces together. Supporting the future development of infodemiology also aligns with broader international goals of creating a more sustainable future for humanity, many outlined in the Goal 3 and Goal 9 health and technology and innovation targets of the UN’s Sustainable Development Goals. Hence, now represents a crucial time for society to leverage “data for good” through the research and practice of infodemiology, leading to the generation of actionable public health intelligence to address current and future global health challenges as they may arise.

## Call to the Infodemiology Community

The primary goal of JMIR Infodemiology is to foster the development of a multistakeholder and multidisciplinary community of researchers, practitioners, and advocates with shared goals of advancing the field of infodemiology to improve health outcomes and tackle other critical social challenges in what is now a digitally intensive era. This includes challenging ourselves to continuously innovate in our methods, including adding new sources of data, conducting more multimodal research, and exploring new methodological approaches to bridge infodemiology with health education, promotion, interventions, and policy. Equally important is the need to generate public health intelligence that is meaningful and actionable, including exploring new content types that the journal will launch in the future that are more responsive to detecting infodemic events and rapidly reporting them to our infodemiology community for real-world impact. Supporting more robust translation and dissemination efforts, the journal will also support more enhanced content such as data visualizations and dashboards that can further augment infodemiology findings. The journal will also purposefully help to ensure adequate representation of neglected health topics and provide dedicated space to discuss important practical, ethical, policy, and e-governance considerations that arise from the evolution of infodemiology and the information ecosystem itself. We invite suggestions for theme issues or special issues, which can be outputs from infodemiology-related conferences and workshops. We welcome authors, reviewers, editors, and other stakeholders who can help us achieve these shared goals of advancing the field of infodemiology and infodemic management, which we agree has—in the words of Chris Zielinski [27]—“a short history, a long future.”

## Abbreviations

ICT: information and communication technology
JMIR: Journal of Medical Internet Research
UN: United Nations
WHO: World Health Organization
